# Early mobilisation in mechanically ventilated patients: a systematic integrative review of definitions and activities

**DOI:** 10.1186/s40560-018-0355-z

**Published:** 2019-01-17

**Authors:** Catherine Clarissa, Lisa Salisbury, Sheila Rodgers, Susanne Kean

**Affiliations:** 1 Department of Nursing Studies, School of Health in Social Science, University of Edinburgh, Medical School, Teviot Place, Edinburgh, EH8 9AG UK; 2 Division of Dietetics, Nutrition and Biological Sciences, Physiotherapy, Podiatry and Radiography, Queen Margaret University, Queen Margaret University Drive, Musselburgh, EH21 6UU UK

**Keywords:** Artificial respiration, Critical illness, Early ambulation, Early mobilisation, Humans, Integrative review, Intensive care unit, Mechanical ventilators, Rehabilitation, Review

## Abstract

**Background:**

Mechanically ventilated patients often develop muscle weakness post-intensive care admission. Current evidence suggests that early mobilisation of these patients can be an effective intervention in improving their outcomes. However, what constitutes early mobilisation in mechanically ventilated patients (EM-MV) remains unclear. We aimed to systematically explore the definitions and activity types of EM-MV in the literature.

**Methods:**

Whittemore and Knafl’s framework guided this review. CINAHL, MEDLINE, EMBASE, PsycINFO, ASSIA, and Cochrane Library were searched to capture studies from 2000 to 2018, combined with hand search of grey literature and reference lists of included studies. The Critical Appraisal Skills Programme tools were used to assess the methodological quality of included studies. Data extraction and quality assessment of studies were performed independently by each reviewer before coming together in sub-groups for discussion and agreement. An inductive and data-driven thematic analysis was undertaken on verbatim extracts of EM-MV definitions and activities in included studies.

**Results:**

Seventy-six studies were included from which four major themes were inferred: (1) *non-standardised definition*, (2) *contextual factors*, (3) *negotiated process* and (4) *collaboration between patients and staff*. The first theme indicates that EM-MV is either not fully defined in studies or when a definition is provided this is not standardised across studies. The remaining themes reflect the diversity of EM-MV activities which depends on patients’ characteristics and ICU settings; the negotiated decision-making process between patients and staff; and their interdependent relationship during the implementation.

**Conclusions:**

This review highlights the absence of an agreed definition and on what constitutes early mobilisation in mechanically ventilated patients. To advance research and practice an agreed and shared definition is a pre-requisite.

## Background

Advances in science, technology and patient care management in the field of intensive care medicine have led to a steady and continuing increase in patients surviving a critical illness episode [1–5]. However, as Herridge [6] highlights *surviving critical illness is not the happy ending that we imagined for our patients*. The reality of post-intensive care creates challenges for patients and families including social recovery, financial burden and adjustments to physical and psychological impairments [7–13]. These long-term difficulties are now referred to as post-intensive care syndrome (PICS) [7, 8].

Mechanically ventilated patients warrant closer attention given the frequent use of mechanical ventilation in ICUs worldwide [14, 15] and risk of patients developing Intensive Care Unit Acquired Weakness (ICU-AW) which is a significant concern in PICS [16–18]. ICU-AW describes a syndrome involving muscle wasting and is associated with higher mortality, poor patient outcomes and a delay of weaning process [19–23].

Early mobilisation while the patient is being mechanically ventilated has been proposed as a promising intervention to counteract ICU-AW, and research suggests it is a safe and feasible intervention [24–26]. The term ‘early mobilisation in mechanically ventilated patients’ (EM-MV) is used interchangeably in the literature and is sometimes referred to as early rehabilitation, early mobility, progressive mobility and early ambulation. While there is some consensus regarding safety criteria to mobilise mechanically ventilated patients [27] and physical rehabilitation for ICU survivors [28], there is currently no unified definition of EM-MV. This lack of definition impacts on the generalisability of studies, their transferability when implementing EM-MV into practice and the conduct of future research. In this current work, we provide a comprehensive and systematic review of the literature to understand how EM-MV is defined and described by different authors. The review questions are as follows:

1. How is early mobilisation in mechanically ventilated patients defined across studies?

2. What types of early mobilisation activities in mechanically ventilated patients are reported in the literature?

## Methods

### Design

**Fig. 1:**
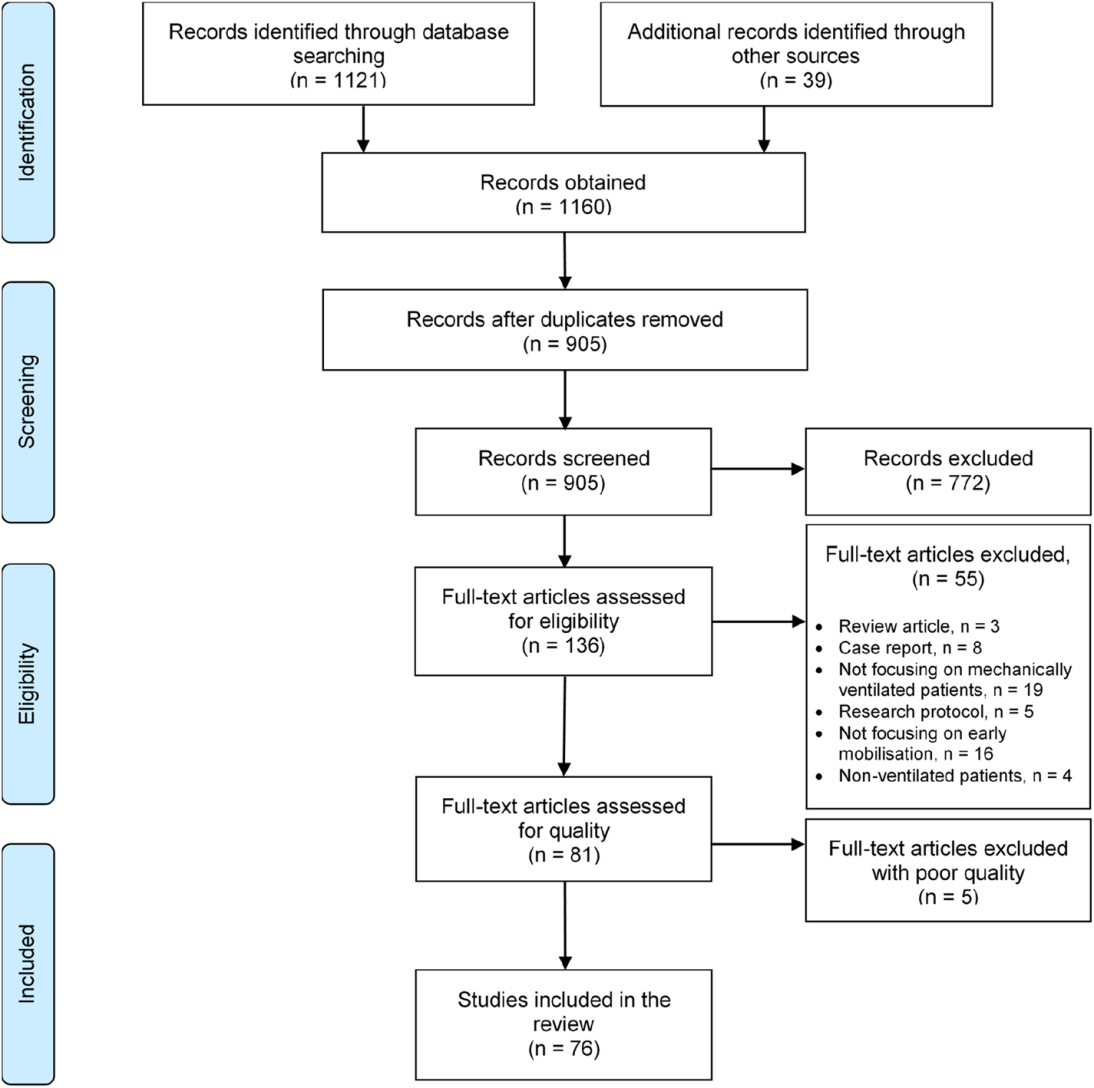
PRISMA flow diagram (PRISMA 2009)

Whittemore and Knafl’s framework [29] guided this review: problem identification, literature search, data evaluation, data analysis and presentation. All quantitative and qualitative designs were included in synthesising the current evidence [29, 30]. The flow diagram of the identified, included and excluded literature is presented using the Preferred Reporting Items for Systematic Reviews and Meta-Analyses [31] (see Fig. 1). The review protocol was registered with PROSPERO International Prospective Register of Systematic Reviews: CRD42016039753 (http://www.crd.york.ac.uk/PROSPERO/display_record.asp?ID=CRD42016039753).

### Search methods

The search strategy was developed in consultation with the University of Edinburgh’s librarian to ensure that we captured all relevant published (peer-reviewed) and unpublished studies (including non-peer-reviewed and grey literature) of EM-MV. Three strategies were used to identify literature: (1) searching six electronic databases: CINAHL, MEDLINE, EMBASE, PsycINFO, ASSIA and Cochrane Library; (2) identifying grey literature by searching: PubMed, Google Scholar, Centre for Reviews and Dissemination (CRD), National Institute for Health and Care Excellence (NICE) and Scottish Intercollegiate Guidelines Network (SIGN); and finally (3) hand-searching reference lists of included studies. Key terms, subject headings and the complete search strategy can be accessed at http://www.crd.york.ac.uk/PROSPEROFILES/39753_STRATEGY_20160819.pdf.

Two review authors (CC, LS) independently screened the title and abstracts for eligibility using our inclusion and exclusion criteria (Table 1). Full-text articles of potential studies were obtained for further assessment. Then, CC and LS had meetings to discuss and compare the results. Disagreements were resolved by discussions with the other reviewers (SR and SK).

**Table 1:** Inclusion and exclusion criteria used in this review

Inclusion criteria	Exclusion criteria
1. Published in English and German. 2. Published between January 2000 and October 2018. 3. Reported on adult patients (aged 18 years and over) receiving early mobilisation while being mechanically ventilated. 4. Measured the outcomes of early mobilisation in mechanically ventilated patients or evaluated the experiences, views and attitudes of mechanically ventilated patients and/or ICU staff; *and either:* 5.1 Reported primary research using a quantitative approach (experimental and/or observational study designs, including randomised controlled trial, case control and cohort study) or any qualitative approach (all study designs). 5.2 Reported secondary research including systematic reviews and meta-analyses.	1. Reported patients aged under 18 years. 2. Reported on patients undergoing early mobilisation without mechanical ventilation support. 3. Evaluated the experiences, views and attitudes of other parties other than ICU staff and/or patients involved in EM-MV. 4. Review articles without a formal search strategy and quality appraisal. 5. Poster or conference proceedings.

*Abbreviations*: *EM-MV* early mobilisation in mechanically ventilated patients*, ICU* intensive care unit

### Quality appraisal and data extraction

We used the Critical Appraisal Skills Programme (CASP) tools [32] to appraise the quality of included studies according to their designs including case control, cohort, randomised controlled trial, systematic review and qualitative [32]. Two screening questions at the beginning of the CASP tools [32] were used to assess the quality of studies to determine their inclusion or exclusion. We used this section as the cut-off points for indicating poor quality and excluded poor-quality studies at this point.

The first author (CC) developed a data extraction form in a Microsoft Office 2016 Excel spreadsheet with the following variables: authors, country of origin, study designs, settings, aim(s), sample size, EM-MV definition and activities*.* Further, the first author (CC) performed the first quality appraisal and data abstraction for all included studies. The studies were then divided into three groups and assigned and reviewed independently by three different review authors (LS, SR, SK) before the first author had individual meetings with each review author. This strategy facilitated the process of comparison between the review authors and agreeing on the quality and extracted data of each study. Any disagreement in a sub-group was arbitrated by a third reviewer beyond their pair. Five studies were excluded after the quality assessment as we agreed that the studies did not pass the first section of CASP tools.

### Data analysis

Thematic analysis is one of the possible analytical approaches for integrated systematic reviews to summarise study findings [33]. We followed Braun and Clarke’s [34] thematic analysis strategies with an inductive and data-driven approach. The two overarching review questions guided the course of data analysis process: (1) ‘How is EM-MV defined across studies?’ and (2) ‘What types of EM-MV activities are reported in the literature?’

Following the quality appraisal and data extraction, all textual descriptions of EM-MV (definitions and activities) stated in the published articles were considered as data and analysed and coded for themes using NVivo11. Each study was read and examined to identify texts and phrases used defining EM-MV or describing EM-MV activities. To explore EM-MV definitions, studies were classified into one of two groups, studies with either full or partial definition of EM-MV. A full EM-MV definition means that the study defines both ‘early’ and ‘mobilisation’ (including their synonyms, for instance, mobility, rehabilitation, ambulation). Studies defining either ‘early’ or ‘mobilisation’ were considered as studies with a partial EM-MV definition. We collated the descriptions of the EM-MV activities from all included studies.

The first author (CC) analysed and coded all obtained verbatim extracts of EM-MV definitions and activities in included studies. Codes were then grouped for similarities and patterns into categories. Each category was given a definition and codes were included in more than one category if relevant. The categories were developed by asking an analytical question: ‘What similarities/patterns do these codes imply?’ The developing data analysis were discussed in regular team meetings. In meetings, we theorised codes and categories into themes and sub-themes by asking a question: ‘What do these codes and categories mean?’ Importantly, the authors’ expertise in critical care nursing (CC, SR, SK) and physiotherapy (LS) provided different professional insights and thus informed the development of themes and sub-themes from different theoretical backgrounds. Regular meetings were continued until all review authors agreed on final themes and sub-themes.

## Results

### Search outcome

Figure 1 (PRISMA flow diagram) details the selection process for inclusion/exclusion of studies in this review. The initial search identified a total of 1160 articles. Removal of duplicates and application of inclusion and exclusion criteria when screening titles and abstracts resulted in 136 studies for inclusion. Full texts of 136 studies were obtained and further assessed against inclusion and exclusion criteria (Table 1). After comparing the screening and quality appraisal results, 76 studies (75 journal articles and one PhD thesis) were found eligible for inclusion in this review. All reasons for exclusions were documented (see Fig. 1)

### Overview of the included papers

Included studies were heterogeneous in study design, setting and country of origin. All characteristics of included studies are summarised in Table 2. Cohort studies were the predominant study design (*n* = 33, 43%), followed by RCTs (*n* = 18, 24%) and case control studies (*n* = 11, 15%). Almost half of the studies (*n* = 35, 46%) were conducted in general ICU settings and about one fifth in medical ICUs (*n* = 16, 21%). Most of the studies originate from the USA (*n* = 27, 36%) and Australia (*n* = 9, 12%) perhaps indicating a current focus on and importance of early mobilisation in these countries. Growing worldwide interest in EM-MV research is evidenced by more than a fourfold increase of published international studies in the last decade from 14 in 2000–2010 to 62 in 2011–2018. Multidisciplinary research collaboration among healthcare professionals including medical, nursing, physiotherapy and respiratory therapy staff was explicit, in that 32 studies (42%) were authored by professionals from two different professional groups and 26 studies (34%) with at least three professional groups*.* EM-MV full definitions were obtained from 15 studies (20%) and partial definitions were identified from 15 studies (20%). The rest of the studies (*n* = 46, 61%) did not provide a definition. All studies provided descriptions of EM-MV activities.

**Table 2:** The characteristics of included studies

Characteristic	Number (*n*)	Percentage (%)*
Study designs		
Case control	11	14.5
Case series	2	2.6
Cohort	33	43.4
Qualitative	8	10.5
Randomised controlled trial	18	23.7
Systematic review	4	5.3
Study settings		
General ICU	35	46.1
Medical ICU	15	19.7
Medical-surgical ICU	4	5.3
Neurological ICU	1	1.3
Post ICU	6	7.9
Respiratory ICU	3	3.9
Surgical ICU	2	2.6
Not applicable (unclear or systematic reviews)	10	13.2
Country of origin		
Australia	9	11.8
Australia and New Zealand	3	3.9
Australia and United Kingdom	1	1.3
Austria, Germany, United States	1	1.3
Belgium	1	1.3
Brazil	5	6.6
China	3	3.9
Canada and United States	1	1.3
France	3	3.9
Germany	1	1.3
Italy	1	1.3
Japan	3	3.9
Sweden	1	1.3
Switzerland	1	1.3
Taiwan	5	6.6
Turkey	1	1.3
United Kingdom	8	10.5
United States	27	35.5
Multiple countries (> 3 countries)	1	1.3
Publication year		
2000–2005	1	1.3
2006–2010	13	17.1
2011–2016	42	55.3
2016–2018	20	26.3
Professional groups of the authors		
All medical staff	7	9.2
All nursing staff	2	2.6
All physiotherapy staff	9	11.8
Combination		
Medical + nursing staff	4	5.3
Medical + occupational therapy staff	1	1.3
Medical + physiotherapy staff	23	30.3
Medical + respiratory therapy staff	1	1.3
Medical + profession unknown	1	1.3
Nursing + physiotherapy staff	1	1.3
Nursing + respiratory therapy staff	1	1.3
Multidisciplinary (> 3 professions)	26	34.2
Definition of EM-MV		
Full definition	15	19.7
Partial definition		
Definition of early	2	2.6
Definition of mobilisation	13	17.1
No definition	46	60.5

*May not be exactly 100% due to rounding

*Abbreviations: EM-MV* early mobilisation in mechanically ventilated patients, *ICU* intensive care unit

### Themes

Following thematic analysis [34], four major themes were developed: (1) *non-standardised definition*, (2) *contextual factors*, (3) *negotiated process* and (4) *collaboration between patients and staff*. The definition of each theme is given in Table 3. The first theme is informed by the full and partial EM-MV definitions extracted from the 30 studies that provided a definition. Themes 2, 3 and 4 are inferred from the descriptions of EM-MV activities from across all included studies.

**Table 3:** Definition of themes inferred in this review

Theme	Definition
Non-standardised definition	Absence of a standard EM-MV definition in the literature.
Contextual factors	Factors relating to patient’s mechanical ventilation status and ICU settings that are taken into account in EM-MV.
Negotiated process	The process of negotiation taken by the key stakeholders of EM-MV (mechanically ventilated patients and staff) in order to actuate EM-MV.
Collaboration between patients and staff	The partnership between mechanically ventilated patients and staff to jointly carry out EM-MV.

*Abbreviations*: *EM-MV* early mobilisation in mechanically ventilated patients, *ICU* intensive care unit

Each theme with the sub-themes and categories is discussed in the following section. Themes, sub-themes and categories are summarised in Table 4 with examples of verbatim extracts to illustrate our interpretations. The theme(s) identified in each study are presented in Table 5.

**Table 4:** Sub-themes, categories and example(s) of verbatim extracts of each theme in included studies

Themes *Sub-themes*	Categories	Example(s) of verbatim extracts and references
Non-standardised definition		
*Practice variation*	Timing of commencement	• *Beginning within 24 h of ICU admission* [42]; • *Within 72 h of mechanical ventilation* [45]; • *Where the patients could assist with the activity using their own muscle strength and control* [48].
	Activities	• *Involved axial loading of the spine and/or long bones *[35, 52]; • *Any activity beyond range of motion* [43].
	Team diversity	• *A program of physiotherapist-directed* [37]; • *Performed by a care provider (nursing, physical or occupational therapy) *[43].
*Expectation of outcome*	Preventative measures of ICU complications	• *To prevent joint contractures* [46];
	Maintaining patient’s mobility	• To maximize physical activity at the highest functional level the patient could achieve [37].
	Improving impairment	• *To induce acute physiological responses (enhancing ventilation, central and peripheral circulation, muscle metabolism, and alertness*) [42]
Contextual factors		
*Mechanical ventilation utilisation*	Intubation types	• *MV was provided to 51% of patients, including 14% with tracheostomy* [42]; • *MV was defined as any ventilation via an endotracheal tube (ETT), tracheostomy tube, or non-invasive positive pressure ventilation* [77].
	Mechanical ventilation duration	• *To initiate the early mobilization program within 72 hours of MV* [87]; • *Occurred while the patient was receiving invasive ventilation* [48].
*ICU context*	ICU stay	• *Continuing through the ICU stay* [24].
	Activity space	• *Mobilizing patients out of bed in the ICU can be seen as an earlier rehabilitation*[64]; • *Both leg and arm exercise with the patient in bed* [75].
	Protocol vs order	• *The early mobilization group (EMG) patients received a systematic early mobilization protocol, twice a day, every day of the week*[46]; • *Activity orders for critically ill patients required a physician orders with all activity performed by either the bedside nurse and/or a physical occupational therapist *[43].
Negotiated process		
*Stakeholder decisions*	Clinical staff judgement	• *The decision to mobilise patients out of bed only after tracheostomy formation is based on the decision that a tracheostomy presents as a stable airway* [51].
	Informed consent	• *The physical therapy intervention started when the informed consent was obtained* [72]; • *Acquire informed consent (e.g., waiting until evening family visits or allowing family members time to think about the decision to enrol)* [49].
*Goal setting*	Progressive mobility	• *The types of functional activities performed during treatment sessions were recorded, including (1) rolling, (2) sitting at the edge of the bed, (3) transferring from sitting to standing, (4) ambulation* [86].
	Improving impairment	• *The 30-minute PT sessions, including abdominal breathing training, respiratory muscle weight training, passive and active joints exercises, upper and lower limb exercises*[79].
	Regaining independence	• *Sitting balance activities were followed by participation in activities of daily living (ADLs) and exercises that encouraged increased independence with functional tasks* [25].
Collaboration between patients and staff		
*Patient participation*	Active	• *Only in 24% of the sessions was more active functional mobilization performed (SOOB, standing, and walking)*[68].
	Passive	• *A combination of passive exercise including positioning, joint range of movement, and hoist transfer to chair* [85].
*Level of assistance*	Independence	• *Patients were first allowed to attempt each activity independently* [26].
	Staff assistance	• *With the assistance of a physical therapist, respiratory therapist and an ICU nurse* [103].

Abbreviations: *ADL* activity of daily living, *EMG* early mobilisation group, *ETT* endotracheal tube, *ICU* intensive care unit, *MP* mobility protocol, *MV* mechanical ventilation, *PT* physiotherapy, *SOOB* sit out of bed, *SPT* standard physical therapy

**Table 5:** A chronological summary of included studies

Author (publication year) *Country*	Study design*	Setting	Study aim(s)	Total sample size (*n*)	Theme**			
					1	2	3	4
Martin et al. (2005) [74] *USA*	Cohort	Post ICU	To evaluate the prevalence and magnitude of weakness in patients receiving chronic mechanical ventilation and the impact of providing aggressive whole-body rehabilitation on conventional weaning variables, muscle strength, and overall functional status.	49		✓	✓	✓
Chiang et al. (2006) [73] *Taiwan*	RCT	Post ICU	To examine the effects of 6 weeks of physical training on the strength of respiratory and limb muscles, on ventilator-free time and on functional status in patients requiring prolonged mechanical ventilation.	32		✓	✓	
Bailey et al. (2007) [24] *USA*	Cohort	Respiratory ICU	To determine whether early physical activity is feasible and safe in respiratory failure patients.	103	✓	✓	✓	✓
Bahadur et al. (2008) [51] *United Kingdom*	Cohort	General ICU	To define the number of occasions of sitting out of bed in patients in the ICU following tracheostomy formation.	30	✓	✓	✓	✓
Morris et al. (2008) [104] *USA*	Cohort	Medical ICU	To assess the frequency of physical therapy, site of initiation of physical therapy and patient outcomes comparing respiratory failure patients who received usual care compared with patients who received physical therapy from a Mobility Team using the mobility protocol.	330		✓	✓	✓
Skinner et al. (2008) [107] *Australia*	Cohort	General ICUs	To identify methods of exercise prescription by physiotherapists across Australian ICUs, including the most commonly used activities for both mechanically ventilated and spontaneously breathing patients; and to determine the outcome measures used for the evaluation of exercise intervention.	111		✓	✓	✓
Thomsen et al. (2008) [38] *USA*	Cohort	Respiratory ICU	To determine whether transfer of respiratory failure patients to the respiratory ICU improved ambulation, independent of the underlying pathophysiology.	104	✓	✓	✓	✓
Malkoç et al. (2009) [105] *Turkey*	Case control	Medical ICU	To evaluate the effect of physiotherapy on ventilator dependency and lengths of ICU stay.	510		✓	✓	
Schweickert et al. (2009) [25] *USA*	RCT	Medical ICU	To assess the efficacy of combining daily interruption of sedation with physical and occupational therapy on functional outcomes in patients receiving mechanical ventilation in intensive care.	104		✓	✓	✓
Bourdin et al. (2010) [64] *France*	Cohort	Medical ICU	To describe the experience in early rehabilitation of ICU patients undergoing mechanical ventilation and its effects on physiologic outcomes.	20		✓	✓	✓
Needham et al. (2010) [103] *USA*	Case control	Medical ICU	To evaluate the effect of the quality improvement project on the number of physical and occupational therapy consultations/treatments and length of stay, in comparison with the prior year.	57		✓	✓	✓
Pohlman et al. (2010) [26] *USA*	Cohort	Medical ICU	To describe a protocol of a daily sedative interruption and early physical and occupational therapy, including neurocognitive state, potential barriers and adverse events related to this intervention.	49		✓	✓	✓
Yang et al. (2010) [79] *Taiwan*	Cohort	Post ICU	To understand the characteristics of ventilator dependence in patients and the potential effects of physical therapy on ventilator weaning and patients’ functional status.	126		✓	✓	✓
Zanni et al. (2010) [86] *USA*	Cohort	Medical ICU	To describe the frequency, physiologic effects, safety and patient outcomes associated with traditional rehabilitation therapy.	32		✓	✓	
Chen et al. (2011) [72] *Taiwan*	RCT	Post ICU	To study the outcomes of functional status, survival rate and ventilator-free status for prolonged mechanical ventilation patients 1 year after physical therapy training enrolment.	34		✓	✓	✓
Clini et al. (2011) [75] *Italy*	Cohort	Respiratory ICU	To assess changes in functional status and whether the degree of functional recovery after a comprehensive rehabilitation program influenced hospital outcomes in a population of tracheostomised and chronically ventilated patients admitted for weaning.	77		✓	✓	✓
Nordon-Craft et al. (2011) [97] *USA*	Case series	N/A	To describe safety and feasibility of participation in physical therapy intervention for individuals with ICU-acquired weakness who required MV for at least 7 days and characterise the examination and intervention procedures with sufficient detail that clinicians can implement a similar strategy.	19		✓	✓	✓
Chen et al. (2012) [96] *Taiwan*	RCT	Post ICU	To evaluate the effects of an exercise training program on pulmonary mechanics, physical functional status and hospitalisation outcomes in terms of respiratory care centre stay, mechanical ventilator weaning rate and mortality rate in patients requiring prolonged mechanical ventilation.	27			✓	
Dantas et al. (2012) [46] *Brazil*	RCT	General ICU	To evaluate the effects of an early mobilisation protocol on respiratory and peripheral muscles.	59	✓	✓	✓	✓
Ronnebaum et al. (2012) [102] *USA*	Case control	General ICU	To compare the effectiveness of two protocols: mobility protocol (MP) and Standard Physical Therapy (SPT) for patients with respiratory failure.	28		✓	✓	✓
Winkelman et al. (2012) [49] *USA*	Case control	Medical-Surgical ICUs	To compare standard care versus an early mobility protocol and to examine the effects of exercise on vital signs and inflammatory biomarkers and the effects of the nurse-initiated mobility protocol on outcomes.	75	✓	✓	✓	✓
Berney et al. (2013) [57] *Australia and New Zealand*	Cohort	General ICUs	To document current physiotherapy mobilisation practices and focus specifically on mobilisation practices in patients requiring prolonged mechanical ventilation, defined as more than 48 h.	498	✓	✓		
Camargo Pires-Neto et al. (2013) [92] *Brazil*	Case Series	Medical ICU	To evaluate the hemodynamic, respiratory and metabolic effects of a cycling exercise performed during the first 72 h of mechanical ventilation.	19		✓	✓	✓
Davis et al. (2013) [36] *USA*	Cohort	Medical-Surgical ICU	To determine the feasibility of employing a standard early mobilisation protocol, while systematically collecting patient mobility data and short-term functional outcomes from critically ill, mechanically ventilated, older adults.	15	✓	✓	✓	✓
Dinglas et al. (2013) [60] *USA*	Cohort	General ICUs	To evaluate the association of patient, ICU and hospital factors with the time to first occupational therapy intervention in the ICU in a prospective cohort of mechanically ventilated patients with acute lung injury.	514	✓		✓	✓
Harrold (2013) [35] *Australia*	Case control	General ICU	To implement a system change that supported safe increases in mobilisation rates of all intensive care patients who were mechanically ventilated for three or more calendar days.	412	✓	✓	✓	✓
Li et al. (2013) [59] *China*	Systematic review	N/A	To investigate the effectiveness and safety of active mobilisation on improving physical function and hospital outcomes in patients undergoing mechanical ventilation for more than 24 h.	17	✓	✓	✓	✓
Mendez-Tellez et al. (2013) [106] *USA*	Cohort	General ICUs	To evaluate the association of patient, ICU and hospital factors with the time to starting physical therapy in a prospective cohort of mechanically ventilated patients with acute lung injury.	503		✓	✓	✓
Williams and Flynn, (2013) [99] *United Kingdom*	Qualitative	N/A	To explore the physiotherapists understanding and experience of implementing early rehabilitation in critically ill patients.	6		✓		
Dinglas et al. (2014) [58] *USA*	Case control	Medical ICU	To evaluate the sustained effect of a quality improvement project on the timing of initiation of active physical therapy intervention in patients with acute lung injury.	243	✓	✓	✓	✓
Dong et al. (2014) [67] *China*	RCT	General ICU	To investigate the feasibility of early rehabilitation therapy in patients with mechanical ventilation.	60		✓	✓	✓
Jolley et al. (2014) [43] *USA*	Cohort	Medical ICU	To assess clinician knowledge regarding early mobilisation and identify barriers to its provision.	120	✓	✓		
Nydahl et al. (2014) [65] *Germany*	Cohort	General ICUs	To undertake a 1-day point-prevalence study of mobilisation of mechanically ventilated patients in ICUs across Germany, including evaluating associations with perceived barriers to mobilisation and complications during mobilisation.	116		✓	✓	
Patel et al. (2014) [45] *USA*	Cohort	Medical ICU	To determine if early mobilisation affects glycaemic control and, in turn, exogenous insulin requirements in critical illness.	104	✓	✓		
Bakhru et al. (2015) [41] *USA*	Cohort	General ICUs	To evaluate the current level of diffusion of early mobilisation practice and examine environmental factors that may influence its practice.	500	✓		✓	✓
Barber et al. (2015) [88] *Australia*	Qualitative	General ICU	To determine the barriers and facilitators of early mobilisation in the ICU.	25		✓		
Berney et al. (2015) [76] *Australia*	Cohort	General ICU	To measure patterns of physical activity in a group of critically ill patients.	41		✓	✓	✓
Collings and Cusack (2015) [85] *United Kingdom*	RCT	General ICU	To quantify and compare the acute physiological response of critically ill patients during a passive chair transfer or a sitting on the edge of the bed.	10		✓	✓	✓
Eakin et al. (2015) [116] *USA*	Qualitative	Medical ICU	To describe a multidisciplinary team perspective regarding how to implement and sustain a successful early rehabilitation programme.	20		✓		
Harrold et al. (2015) [52] *Australia and United Kingdom*	Cohort	General ICUs	To evaluate baseline practise and the perceived barriers to early mobilisation in ICU across multiple sites in two different countries with different systems of health care delivery.	830	✓	✓		✓
Holdsworth et al. (2015) [61] *Australia*	Qualitative	General ICU	To elicit attitudinal, normative, and control beliefs towards mobilising ventilated patients in the ICU to generate items for a second-phase questionnaire and inform the development of a tailored implementation intervention.	22	✓	✓	✓	✓
Jolley et al. (2015) [100] *USA*	Cohort	General ICUs	To determine what proportion of hospitals caring for mechanically ventilated patients across Washington State use physical activity in the ICU and to identify process of care factors associated with reported activity delivery.	47		✓	✓	✓
Kayambu et al. (2015) [95] *Australia*	RCT	General ICU	To determine whether early physical rehabilitation improves physical function and associated outcomes in patients with sepsis.	50		✓	✓	✓
McWilliams et al. (2015) [66] *United Kingdom*	Case control	General ICU	To evaluate the impact of an early and enhanced rehabilitation programme for mechanically ventilated patients in a large tertiary referral mixed-population ICU.	582		✓	✓	✓
Ota et al. (2015) [47] *Japan*	Case control	General ICU	To clarify the benefits of early mobilisation for mechanically ventilated patients for their survival to discharge to home from the hospital.	108	✓	✓	✓	✓
Camargo Pires-Neto et al. (2015) [68] *Brazil*	Cohort	Medical ICU	To characterise the provision of early mobilisation therapy in critically ill patients in a Brazilian medical ICU and to investigate the relationship between physical activity level and clinical outcomes.	120		✓	✓	✓
Skinner et al. (2015) [69] *Australia*	Cohort	General ICU	To report the incidence of usual care physiotherapy, specifically treatment and modalities used, in a sample of subjects admitted to a single tertiary Australian ICU.	100		✓	✓	✓
The TEAM Study Investigators (2015) [48] *Australia and New Zealand*	Cohort	General ICUs	To investigate current mobilisation practice, strength at ICU discharge and functional recovery at 6 months among mechanically ventilated patients.	192	✓	✓	✓	✓
Toccolini et al. (2015) [70] *Brazil*	Cohort	General ICU	To assess the effects of passive orthostatism on various clinicophysiologic parameters of adult ICU patients, by daily placement on a tilt table.	23		✓		✓
Witcher et al. (2015) [71] *USA*	Case control	Neurological ICU	To examine the effect of an early mobilisation protocol on sedation practices of critically ill, mechanically ventilated patients.	68		✓	✓	✓
Bakhru et al. (2016) [56] *France, Germany, United Kingdom and USA*	Cohort	General ICUs	To evaluate organisational characteristics that enable early mobilisation practice.	951	✓	✓	✓	
Dong et al. (2016) [93] *China*	RCT	General ICU	To evaluate the influence of early rehabilitation therapy on patients with more than 72 h of prolonged mechanical ventilation after coronary artery bypass surgery.	106		✓	✓	✓
Hickmann et al. (2016) [42] *Belgium*	Cohort	General ICU	To demonstrate that early mobilisation performed within the first 24 h of ICU admission proves to be feasible and well tolerated in the vast majority of clinically ill patients.	171	✓	✓	✓	✓
Hodgson et al. (2016) [37] *Australia and New Zealand*	RCT	General ICUs	To determine if the early goal-directed mobilisation intervention could be delivered to patients receiving mechanical ventilation with increased maximal levels of activity compared with standard care.	50	✓	✓	✓	✓
Morris et al. (2016) [84] *USA*	RCT	Medical ICU	To compare standardised rehabilitation therapy to usual ICU care in acute respiratory failure	300		✓	✓	✓
Schaller et al. (2016) [101] *Austria, Germany and USA*	RCT	Surgical ICUs	To test if early, goal-directed mobilisation, using a strict mobilisation algorithm combined with facilitated inter-professional communication leads to improved mobility during admission, decreased length of stay, and increased functional independence at hospital discharge.	200		✓	✓	✓
Curtis and Irwin (2017) [50] *United Kingdom*	Qualitative	N/A	To increase understanding of nurses’ perspectives on ambulating mechanically ventilated patients, and to determine why this is not a routine part of ICU patient care.	8	✓		✓	✓
Dunn et al. (2017) [62] *USA*	Systematic review	N/A	To evaluate the strength of existing publications to determine if active mobilisation interventions in prolonged mechanical ventilation patients improves physical function, ventilator weaning rates, pulmonary mechanics, and clinical hospital outcomes such as length of stay and mortality.	8	✓			
Jolley et al. (2017) [77] *USA*	Cohort	General ICU	To determine the prevalence and character of mobility for ICU patients with acute respiratory failure.	42		✓	✓	✓
Lai et al. (2017) [87] *Taiwan*	Case control	Medical ICU	To evaluate the effects of a quality improvement programme to introduce early mobilisation on the outcomes of patients with mechanical ventilation in the ICU.	153		✓	✓	✓
McWilliams et al. (2017) [39] *United Kingdom*	Case control	General ICUs	To investigate whether the Sara Combilizer® could facilitate safe and early mobilisation of critically ill patients at high risk of ICU-acquired weakness who would otherwise be unable to get out of bed, thereby reducing time to first mobilisation.	63	✓	✓	✓	✓
Parry et al. (2017) [98] *Australia*	Qualitative	General ICU	To identify the barriers and enablers that influence clinicians’ implementation of early rehabilitation in critical care.	26		✓	✓	✓
Sibilla et al. (2017) [78] *Switzerland*	Cohort	General ICUs	To characterise the highest level of mobilisation achieved in mechanically ventilated patients as defined by the valid and reliable ICU Mobility Scale and to characterise the potential safety events related to mobilisation and perceived barriers to mobilisation.	161		✓	✓	✓
Weeks et al. (2017) [44] *USA*	Cohort	Medical-Surgical ICU	To investigate the feasibility of early mobilisation and describe the rehabilitation interventions and functional discharge outcomes in critically ill patients with cancer.	42	✓	✓	✓	✓
de Queiroz et al. (2018) [63] *Brazil*	Systematic review	N/A	To evaluate of the description of the active mobilisation protocols in patients on invasive mechanical ventilation at ICUs.	17	✓	✓		✓
Goddard et al. (2018) [81] *Canada and USA*	Qualitative	N/A	To explore barriers and facilitators to early rehabilitation for critically ill patients receiving invasive mechanical ventilation.	40		✓		
Liu et al. (2018) [83] *Japan*	Cohort	General ICU	To investigate the safety of early mobilisation according to the Maebashi Early Mobilisation protocol conducted by ICU physicians.	72		✓	✓	✓
McWilliams et al. (2018) [40] *United Kingdom*	RCT	N/A	To explore the feasibility of earlier and enhanced rehabilitation for patients mechanically ventilated for ≥5 days and to assess the impact on possible long-term outcome measures for use in a definitive trial.	102	✓	✓	✓	✓
Medrinal et al. (2018) [90] *France*	RCT	N/A	To compare the physiological effects of four common types of bed exercise in intubated, sedated patients confined to bed in the ICU, in order to determine which was the most intensive.	19		✓	✓	✓
Phelan et al. (2018) [55] *Australia*	Systematic review	N/A	To identify the key factors that underpin successful implementation and sustainability of early mobilisation in adult intensive care units.	13	✓		✓	✓
Ringdal et al. (2018) [91] *Sweden*	Qualitative	General ICUs	To explore patient recollections and experiences of early mobilisation, including in-bed cycling.	11		✓	✓	✓
Sarfati et al. (2018) [80] *France*	RCT	Surgical ICU	To investigate whether cardiothoracic surgery patients expected to require prolonged ICU management benefited from the addition of daily tilting to an early mobilisation program.	125		✓	✓	✓
Taito et al. (2018) [54] *Japan*	Cohort	General ICUs	To clarify intensive care unit-level factors facilitating out-of-bed mobilisation in mechanically ventilated patients with orotracheal tubes.	168		✓	✓	✓
Verceles et al. (2018) [53] *USA*	RCT	Post ICU	To compare the effects of adding a progressive multimodal rehabilitation program to usual care.	32	✓	✓	✓	✓
Winkelman et al. (2018) [94] *USA*	RCT	General ICUs	To examine whether the delivered intervention influenced inflammatory serum markers and to explore whether the dose of the delivered intervention influenced patient outcomes.	54		✓	✓	✓
Wright et al. (2018) [82] *United Kingdom*	RCT	Medical-surgical ICU	To evaluate the effects of two different intensities of early rehabilitation therapy - intensive versus standard - on the recovery of physical health-related quality of life at 6 months.	308		✓	✓	✓

*Based on CASP tools

**1 non-standardised definition, 2 contextual factors, 3 negotiated process, 4 collaboration between patients and staff

Abbreviations: *CASP* Critical Appraisal Skills Programme, *ICU* intensive care unit, *MP* mobility protocol, *N/A* not applicable (unclear or systematic reviews), *SPT* standard physical therapy, *RCT* randomised controlled trial

### Theme 1: Non-standardised definition

The first theme, and the key insight of this review, relates to the absence of a standardised EM-MV definition across all included studies. A full definition of EM-MV was evident in 15 of 76 studies [24, 35–48]. A partial definition of EM-MV was provided in 15 studies with two studies defining ‘early’ [49, 50] and 13 studies defining ‘mobilisation’ [51–63]. A total of 46 studies did not provide a definition. From the 30 studies with full and partial definitions of EM-MV, we identified two recurring sub-themes reflecting the different ways that EM-MV was defined: (1) practice variation and (2) expectation of outcome.

#### Sub-theme 1.1: Practice variation

*Practice variation* is defined as diversity of delivery that existed among EM-MV definitions and includes the timing of commencement, the activities and the care team. Most studies regarded any mobilisation activity as early if it is commenced any time during the course of mechanical ventilation [36, 48] or between 48 and 72 h of starting mechanical ventilation [43–45, 47]. Other authors used ICU length of stay to refer to ‘early’ as either 24 h after admission [42], below 14 days length of stay [49] or throughout the ICU stay [24, 38]. EM-MV commencement time was also reported in a non-time-bound manner including any period of time [51], during the recovery [39, 50] or acute stage of illness [40], patient’s ability to engage with the activities [36, 48] and the point at which the patients were deemed stable physiologically [24, 35, 36, 38, 50] and psychologically [50].

Twenty one of the 30 studies incorporated a description of activities in their definition by listing included and excluded activities or providing general descriptions of activities. Most of the studies reported an explicit list of included activities such as cycle ergometry exercises [58, 59, 63], sitting on the edge of bed [24, 35, 38–40, 52, 59, 61], sitting out of bed (in a chair) [24, 35, 38–40, 51, 52, 55, 57, 59], standing using a tilt table [35, 39, 52], standing [35, 39, 40, 52, 55, 59], marching [61] and walking [24, 35, 38–40, 52, 55, 57, 59, 61, 63]. The general descriptions of the activities were exercises involving axial loading exercises [35, 52], movements against gravity [35, 52, 61], active activities [37, 48, 55, 58, 59, 63] and activities requiring energy expenditure of patients [62]. ‘Active’ was indicated in the EM-MV definitions as patients having muscle strength and an ability to control the activities [48], a conscious muscle activation (except breathing) [63] and as certain types of activities such as activities with physiological benefits [55], strengthening and mobility exercise [58] and assisted exercise [59].

Several studies included the details of the care team in their EM-MV definitions. The team was diverse and comprised of clinical and non-clinical staff. The clinical staff involved were physiotherapists (PTs) [24, 37, 43, 44], occupational therapists (OTs) [43, 44], respiratory therapists (RTs) [24, 44] and nurses [24, 43, 44]. The non-clinical staff were technicians [24]. PTs and OTs were reported as key professional groups in evaluating a patient’s readiness for EM-MV [37, 44].

#### Sub-theme 1.2: Expectation of outcome

*Expectation of outcome* reflects the descriptions of the desired effects of EM-MV including preventing ICU complications, maintaining patient’s mobility and improving impairment. Two studies referred to specific preventions such as joint contractures [46] and delirium [44], and one study referred to general prevention which was to counteract immobilisation [62]. Patient’s mobility was targeted at achieving the highest functional level or regaining the functional status before ICU admission [37, 41, 44, 53, 56]. The expected responses of EM-MV in improving impairment were stated in the definitions by describing affected body systems including muscular, respiratory, circulatory and nervous systems [42, 46, 53].

In summary, EM-MV is either not fully defined in studies or when a definition is provided this is not standardised across studies. In the 15 of 76 studies which provided a full definition of EM-MV, there was no standardised EM-MV definition. The sub-themes *practice variation* and *expectation of outcomes* identify how the definitions differed between authors and reflect the main features of EM-MV definitions found in included studies.

### Theme 2: Contextual factors

The theme *contextual factors* encompass the aspects of mechanical ventilation use and the context of ICU settings in the course of EM-MV. This theme was evident in almost all studies (see Table 5) and consists of two sub-themes: (1) mechanical ventilation utilisation and (2) ICU context.

#### Sub-theme 2.1: Mechanical ventilation utilisation

*Mechanical ventilation utilisation* is associated with the type of intubation patients received and the duration of ventilation support while undertaking EM-MV. Forty one of 76 included studies provided the information on intubation type in patients undertaking EM-MV activities. Patients using tracheostomy undertaking EM-MV were reported in 33 studies [24, 36–40, 42, 48, 51, 52, 57, 61, 63–83]. The use of endotracheal tube (ETT) during EM-MV activities was reported in 32 studies [24, 35–40, 47, 48, 52, 54, 57, 61, 63, 65–71, 77, 78, 80–88]. Patients undertaking EM-MV activities with non-invasive ventilation (NIV) was only evident in six studies [65, 77, 78, 80, 82, 89].

EM-MV activities were reported taking place during mechanical ventilation with two apparent categories of duration, namely short term and long term. The short-term duration was described as within 48 h [25, 43, 52, 58, 84, 90, 91], within 72 h [44, 45, 87, 92] or after 48–72 h [57, 80, 82, 90, 93–95]. Long-term duration was beyond 7–21 days [39, 40, 47, 53, 72, 73, 75, 96, 97].

#### Sub-theme 2.2: ICU context

*ICU context* refers literally to the ICU setting and includes the use of protocol or order to initiate EM activities. EM-MV activities were initiated during ICU stay in different timeframes: as soon as possible after admission [74, 98, 99], between 24 and 48 h after admission [42, 75], before 14 days of admission [49] and throughout admission [24, 25, 36, 38, 46, 51, 56, 98–100]. ‘In-bed’ or ‘out-of-bed’ captures different locations in which EM-MV happened (Table 6).

**Table 6:** Reported in-bed and out-of-bed activities in included studies

In-bed activity	Out-of-bed activity
Range of motion [39, 49, 77, 90, 101]	Bed-transfer training [102, 107]
Bridging [37]	Sitting at the edge of bed [54, 57, 77]
Turning [36, 44, 73]	Sitting in a chair [49, 51, 65, 77],
Transferring [25]	Standing [49, 65, 77]
Limb exercise [44, 75]	Marching [53, 65, 77]
Self-care activities [44]	Ambulating [24, 26, 35, 37, 38, 41, 42, 44, 46, 48, 56, 58, 61, 64–69, 71, 72, 76–79, 85, 93, 100–103, 107]
Breathing exercise [90]	
Electrical stimulation [90]	
Sitting in bed [25, 39, 46, 71, 76, 78, 83]	
Sitting at the edge of bed [53, 71]	
Cycling [42, 53, 83, 90, 91]	

Several studies reported that EM-MV was initiated using a protocol or an order. EM-MV was automatically triggered by a protocol to initiate activities following patients’ admissions to ICU in 31 studies [24–26, 35–40, 42, 46, 49, 58, 60, 66, 67, 72, 74–76, 80, 83, 84, 87, 93, 94, 101–105]. Across the studies reporting the requirement of a formal order to initiate EM-MV, staff prescribing the order varied from physicians [43, 47, 64, 79, 86], PTs [48, 51, 68, 85], PTs and OTs [44, 71] to the care team [45].

To summarise, the overall categories and sub-themes encompassed within theme *contextual factors* suggest that EM-MV activities are contextual depending on patient’s mechanical ventilation status, the setting of ICU where EM-MV takes place and the use of a protocol or an order for initiating EM-MV. The findings highlight diverse contexts and inconsistency in EM-MV provision across included studies.

### Theme 3: Negotiated process

*Negotiated process* is concerned with the negotiation occurring between mechanically ventilated patients and staff as stakeholders to bring about EM-MV. This theme has two sub-themes: (1) stakeholder decisions and (2) goal setting.

#### Sub-theme 3.1: Stakeholder decisions

*Stakeholder decisions* refers to factors relating to staff and patients affecting the decision-making process to initiate EM-MV including clinical staff judgement and informed consent given by the patients or their proxies. The staff judgement was related to the assessment of patient safety in undertaking EM-MV and based on patient’s physiological status [24, 35, 36, 38, 49, 87, 103], level of consciousness [26, 71, 98, 103], patient compliance [98] and an established tracheostomy as a sign of a stable airway [51]. Level of consciousness ranged from alert and cooperative patients [98] to those that were delirious based on the Confusion Assessment Method for the Intensive Care Unit (CAM-ICU) [26]. The tools for measuring the level of consciousness were Richmond Agitation-Sedation Scale (RASS) [103] and Glasgow Coma Scale (GCS) [71]. Patients with RASS ≥ − 3 [103] or GCS ≤ 8 [71] were considered as comatose and excluded from the EM-MV activities. Most studies reported that informed consent was sought before commencing EM-MV from the patients or their proxies. In some cases, it was argued that informed consent was not required because EM-MV was part of routine care [24, 35, 38, 42, 44, 51, 52, 60, 64, 66, 74, 76, 80, 86, 102–104].

#### Sub-theme 3.2: Goal setting

*Goal setting* is the sub-theme associated with the treatment aims of EM-MV activities delivered to mechanically ventilated patients and evident across the literature. The goals include (1) progressive mobility, (2) improving impairment and (3) regaining independence. The activities related to each goal are detailed in Table 7.

**Table 7:** Reported treatment goals and activities in included studies

Goal	Type of activities	References
Progressive mobility	Positioning	[36, 46, 47, 50, 65–69, 73, 85, 104, 105]
	Bed head elevation	[41, 47, 65, 67, 93]
	Sitting*	
	Sitting in bed	[25, 37, 41, 46, 54, 58, 65, 74, 76, 78, 87, 93, 103, 104]
	Sitting at the edge of bed	[24–26, 35–39, 41, 44, 46, 51, 52, 55, 57–59, 61, 65–68, 71, 74, 76–78, 82, 83, 85–87, 93, 94, 97, 100, 101, 103, 104, 107]
	Sitting out of bed	[24, 26, 35–39, 42, 44, 46, 49–52, 54, 58, 59, 64–68, 72, 75–78, 85, 87, 93, 95, 100, 103, 104]
	Standing	[26, 35, 36, 42–47, 50, 54, 55, 63, 65–69, 71–80, 82, 83, 85, 88, 93, 94, 97, 101–104]
	Ambulating	[24–26, 35, 37–39, 41, 42, 44, 46, 48–50, 54, 56, 58, 61, 64–69, 71, 72, 74–80, 82, 83, 85, 86, 89, 93–95, 100–103, 107]
Improving impairment	Respiratory system	
	Breathing exercises	[36, 44, 47, 54, 69, 72, 79, 97, 105, 106]
	Muscles and joints	
	Range of motions	[26, 36, 41, 49, 54, 60, 71, 73, 76, 77, 79, 85, 97, 101, 102, 104, 106]
	Limb exercises	[36, 44, 47, 48, 60, 68, 75, 76, 79, 87]
	Strengthening	[53, 58, 72, 73, 82, 86, 96, 98]
	Stretching	[36, 46, 68, 86, 96]
	Counter-resistance	[36, 42, 46, 59, 68, 72–75, 79, 89, 104]
	Weight bearing	[26, 35, 41]
	Cycling	[41, 42, 46, 53, 54, 58, 59, 74, 75, 83, 90–92, 96]
Regaining independence	Transfer training	[25, 26, 39, 44, 46, 53, 55, 61, 69, 73–78, 83, 89, 93–95, 97, 100, 107]
	Marching	[25, 26, 36, 53, 61, 65, 69, 74, 76–78, 83, 89, 94, 97, 101, 107]
	Balance training	[25, 26, 36, 37, 46, 53, 86, 89, 104]
	Activity of daily livings	[25, 26, 36, 44, 60, 86]
	Rolling	[26, 36, 44, 48, 65, 86, 97]
	Bridging	[36, 48]
	Staircase exercises	[74]
	Sitting	see* (progressive mobility)

The progressive mobility reflects the progression of mobility achieved by the patients in EM-MV over time. Mobility progression was phased starting with positioning (*n* = 13) followed by elevating the head of the bed (*n* = 5) and sitting which was further divided into with three stages: (1) sitting in bed (*n* = 14), (2) sitting without back support or at the edge of the bed (*n* = 40) and (3) sitting out of bed (*n* = 34). Progression following on from sitting was standing (*n* = 39) with ambulation (*n* = 46) being the highest level of mobility and which was explicitly stated as the primary goal of EM-MV in some studies [24, 38, 86].

The second goal relates to improving impairment which is concerned with patients’ homeostasis, particularly the functionality of the respiratory system and muscle and joint strength because of EM-MV activities. Breathing exercises were the most commonly reported respiratory-related activity (*n* = 10). EM-MV activities aiming at muscles and joints consisted of a variety of exercises such as ROM (*n* = 17), limbs exercises (*n* = 10), strengthening (*n* = 8), stretching (*n* = 5), counter-resistance (*n* = 12), weight bearing (*n* = 3), and cycling (*n* = 13).

The goal of regaining independence is related to EM-MV activities aiming at preparing the patients for their life after hospital discharge and consisted of functional exercises. Commonly identified exercises were transfer training (*n* = 23), marching (*n* = 17), balance training (*n* = 9), activity of daily livings (ADLs) (*n* = 6), rolling (*n* = 7), bridging (*n* = 2), staircase exercises (*n* = 1) and sitting (sitting in bed, *n* = 14; sitting at the edge of bed, *n* = 40; sitting out of bed, *n* = 34).

In summary, the theme *negotiated process* suggests that the implementation of EM-MV is a result of negotiations between mechanically ventilated patients and staff. Decision-making of staff around whether or not the patient is safe to undertake EM-MV and what type of activities are appropriate with a view of setting a goal was prevalent in the literature. In most studies, EM-MV was usually initiated by a clinical order or by protocol. The requirement of informed consent from the patient or their proxy to commence EM-MV was varied, and consent was not sought if EM-MV was part of routine care.

### Theme 4: Collaboration between patients and staff

The theme *collaboration between patients and staff* refers to the interdependent relationship between mechanically ventilated patients and staff as the stakeholders suggesting that EM-MV requires involvement of both to succeed. The theme is based on two sub-themes: (1) patient participation and (2) level of assistance.

#### Sub-theme 4.1: Patient participation

*Patient participation* describes the degree of active or passive involvement in EM-MV activities. The same activities were not consistently classified as active or passive across all studies. For example, head up position was considered as a passive activity in one study [41], but was viewed as active in another study [67]. Similarly, ROM could be an active [25, 26, 36, 41, 49, 63, 71, 75, 79, 80, 87, 95, 97] or passive activity [26, 36, 41, 46, 47, 49, 54, 59, 60, 71, 74, 77, 79, 80, 85, 87, 90, 95, 97, 101, 104, 106]. Other commonly reported passive activities across studies were in-bed positioning [59, 66, 85] and transfer to a chair with assistance [42, 77, 78, 85]. Assistance was required in non-specific active activities [25, 37, 42, 46, 48, 107] or specific active activities such as ROM [26, 41]. A further important aspect of EM-MV was patient’s ability to interact with staff [87, 98]. Consequently, passivity was described as being associated with the unconscious, sedated and paralysed patients [66, 87].

#### Sub-theme 4.2: Level of assistance

*Level of assistance* refers to the level of support mechanically ventilated patients require when undertaking EM-MV activities. Patients may undertake activities independently or while being supported by staff or in combination with equipment. The most commonly used equipment were a tilt table [35, 39, 42, 52, 64, 66, 69, 70, 80, 82] and walking aids [24, 36, 38, 75, 103]. Several authors reported that assisting a mechanically ventilated patient to mobilise required support between one to four people [24, 48, 93, 103]. Staff members included nurses, OTs, physicians, PTs and RTs [24, 48, 87, 93, 100, 103] while non-clinicians included visiting family members [87] and technicians [24]. Thirteen studies mentioned that patients could perform EM-MV activities independently without the support of staff including sitting and walking [24–26, 35–39, 48, 55, 59, 93, 102].

Overall, the sub-themes *patient participation* and *level of assistance* reflect the collaboration between mechanically ventilated patients and staff to actuate EM-MV activities. What constitutes active or passive about patient participation remains inconclusive as there were some overlaps of interpretations across included studies. The descriptions provided by included studies about the level of assistance required by the patients either the physical support from staff or the use of equipment were scarce and inadequate to conclude the meaning of independent in EM-MV.

## Discussion

It is evident from this systematic review that a definition for EM-MV remains far from being agreed and that EM-MV activities are poorly understood. Our analysis of EM-MV definitions in the literature suggests that EM-MV is both broadly and narrowly defined and thus is problematic for advancing research and practice. The broader definitions are heterogeneous with a vast scope of EM-MV. In contrast, while narrow definitions are desirable in improving validity and reliability in scientific research, we suggest that the variability in, for example, timing and various EM activities, challenges the transferability of study results.

The inconsistency in both broad and narrow definitions raises an issue of comparability between studies and weakens the evidence base for clinicians at the bedside. Questions such as ‘When should we start mobilising our patients?’ and ‘Which activities should we choose?’ are therefore difficult to answer. Researchers should provide a detailed report of timing of EM-MV initiation and details of activities in their research since transparency on these details will promote the uptake of research evidence into practice [108–110].

Regardless of the existence or non-existence of a EM-MV definition in a given study, most included studies have reported the initiation time of EM-MV in relation to mechanical ventilation duration or the length of ICU stay which varied considerably. This variation is an issue of interest and has been previously highlighted by researchers [35, 40, 56]. Bakhru et al. [56], for example, deliberately stated that they did not define ‘early’ due to there being no consensus. Harrold [35] conducted a systematic review to explore timing and activities of EM-MV and predetermined the classifications of timing into three criteria: (1) in ICU with mechanical ventilation, (2) in ICU without mechanical ventilation and (3) not in ICU with no information on mechanical ventilation. Given the rapid onset of muscle wasting within hours of mechanical ventilation [111–114], we believe that Harrold’s [35] classification still appears to be too broad. We suggest that research should be focused on the optimal EM-MV initiation timing after a patient is mechanically ventilated.

The interchangeable use of EM-MV terminology requires some reflection and agreement for consistency. Despite no formal count of verb frequency in our work, we noticed that ‘early mobilisation’ was the most frequently used term. Other terms were ‘early activity’, ‘early exercise’, ‘early mobility’, ‘early occupational/physical therapy’ and ‘early rehabilitation’. We found that studies originating in the USA commonly use the term ‘mobilisation’, whereas in the UK and Europe authors often use the term ‘rehabilitation’. This inconsistency was also evident in individual studies which frequently used terminology interchangeably in their published work. It is not unreasonable to assume that readers may think that different terminologies are referring to different concepts. For example, the studies referring to EM-MV as ‘early rehabilitation’ seem to focus on functional activities such as bridging and ADLs. Studies focusing on ‘early mobility’ or ‘early mobilisation’ tend towards stepwise mobility activities including sitting, standing and ambulation. Understanding and defining what ‘mobilisation’ and ‘rehabilitation’ imply across the international community might be one step in clarifying the conundrum of varied EM-MV terminologies. These differences of terminologies may reflect differing views of researchers and emphasise the absence of a standardised definition of EM-MV.

Our findings show that EM-MV was commonly delivered by a team consisting of clinical and non-clinical staff. The multidisciplinary of EM-MV is reflected by the authors of included studies ranging from medical staff, nursing, PT, OT to RT either as individual or as multidisciplinary author(s). This is an important point since different disciplinary background will impact on how EM-MV is defined and implemented. Future research needs to pay attention to this aspect to maximise insights from different professional backgrounds.

### Review strengths and limitations

A major strength of this review is that the analysis was conducted inductively with transparent documentation at each stage. Thus, the sub-themes and themes inherent in the definitions and activities of EM-MV are based on the existing literature without imposing preconceptions and assumptions of the authors. Furthermore, this review included both primary and secondary studies with a range of objectives. Therefore, it offers broad coverage of literature in this area. The different professional perspectives (nursing and physiotherapy) is another strength of this review since our professional definitions of what exactly constitutes mobilisation varied, and this was reflected both in the research reviewed and in current multidisciplinary ICU care. Finally, the review provides insights into the aspects of EM-MV definition and activities lacking consensus, as demonstrated by conflicting perspectives of authors.

Two potential limitations are apparent in this study. The diverse terminology used around EM-MV in the literature may be a hindrance in capturing all relevant articles. Additionally, this review only included studies in English and German as these are the primary languages of the authors. This restriction may have missed studies published in other languages. However, attempts have been made to minimise this limitation by including multiple databases in the search strategy combined with hand searching of the grey literature and the reference lists of included studies.

### Implications for future research

The findings of this review substantiate the need for an agreed definition and terminology of EM-MV. If we want to promote evidence-based practice, researchers need to speak the same language about what EM-MV is. The absence of a consensus may impede the implementation of evidence-based practice on this topic [115]. The inconsistency of EM-MV terminology may become a complicating matter in EM-MV definitions. We believe that the agreement of terminology used to refer EM-MV is a stepping-stone to moving forward into a clear and consistent definition. We strongly recommend that ICU experts reach consensus on a formal and consistent definition of EM-MV.

Furthermore, the sub-themes and themes that were identified in this review provide a strong base to understand current underlying conceptualisation of EM-MV which could inform the construction of a standardised definition and the type of activities that are considered as EM-MV. Recognising the importance of detailed reporting of research for the purpose of allowing study replication and promoting research evidence uptake into practice [108–110], our results can also be used as a guideline for the details to include in reporting research related to EM-MV.

Most included studies adopted quantitative approaches in investigating EM-MV (see Table 2). Considering that qualitative research could contribute to the insights into effective EM-MV delivery, this review highlights the urgency of the need for more qualitative studies. Some studies have attempted to explore the clinician’s perceptions of EM-MV [50, 61, 81, 88, 98, 99, 116], yet research into patients’ views of EM-MV is lacking as evidenced by only one study found in this review [91]. Exploring patient views is essential as they are the primary participants in EM-MV. Rigorous qualitative research should be developed to facilitate the design of EM-MV as a complex intervention that is aligned with patient and staff expectations [117, 118]. EM-MV practice could then be optimised and promote improved outcomes for patients.

## Conclusion

This review highlights the varied definitions of EM-MV and the necessity for an agreed EM-MV terminology and definition based on consensus and a deeper understanding of what activities constitute EM-MV. This lack of consistency complicates the benchmarking or comparison of results across studies which further hinders the translation of evidence into practice as well as study replication in other settings. A mutual understanding of EM-MV including the terminology, the definition and the constituting activities is required to advance research and to trigger a further discussion on this topic.

## Abbreviations

ADL: Activity of daily living
CAM-ICU: Confusion Assessment Method for the Intensive Care Unit
CASP: Critical Appraisal Skills Programme
CRD: Centre for Reviews and Disseminations
EM-MV: Early mobilisation in mechanically ventilated patients
GCS: Glasgow Coma Scale
ICU: Intensive care unit
ICU-AW: Intensive Care Unit-Acquired Weakness
NICE: National Institute for Health and Care Excellence
NIV: Non-invasive ventilation
OT: Occupational Therapist
PICS: Post-intensive care syndrome
PRISMA: Preferred Reporting Items for Systematic Reviews and Meta-Analyses
PROSPERO: International prospective register of systematic reviews
PT: Physiotherapist
RASS: Richmond Agitation-Sedation Scale
ROM: Range of motion
RT: Respiratory therapist
SIGN: Scottish Intercollegiate Guidelines Network
